# Safe and Robust Mobile Robot Navigation in Uneven Indoor Environments

**DOI:** 10.3390/s19132993

**Published:** 2019-07-07

**Authors:** Chaoqun Wang, Jiankun Wang, Chenming Li, Danny Ho, Jiyu Cheng, Tingfang Yan, Lili Meng, Max Q.-H. Meng

**Affiliations:** 1 Department of Electronic Engineering, The Chinese University of Hong Kong, Hong Kong, China; 2 Computer Science Department, University of British Columbia, Vancouver, BC V6T 1Z4, Canada

**Keywords:** traversable map, regression forest, image-based localization, path planning and navigation

## Abstract

Complex environments pose great challenges for autonomous mobile robot navigation. In this study, we address the problem of autonomous navigation in 3D environments with staircases and slopes. An integrated system for safe mobile robot navigation in 3D complex environments is presented and both the perception and navigation capabilities are incorporated into the modular and reusable framework. Firstly, to distinguish the slope from the staircase in the environment, the robot builds a 3D OctoMap of the environment with a novel Simultaneously Localization and Mapping (SLAM) framework using the information of wheel odometry, a 2D laser scanner, and an RGB-D camera. Then, we introduce the traversable map, which is generated by the multi-layer 2D maps extracted from the 3D OctoMap. This traversable map serves as the input for autonomous navigation when the robot faces slopes and staircases. Moreover, to enable robust robot navigation in 3D environments, a novel camera re-localization method based on regression forest towards stable 3D localization is incorporated into this framework. In addition, we utilize a variable step size Rapidly-exploring Random Tree (RRT) method which can adjust the exploring step size automatically without tuning this parameter manually according to the environment, so that the navigation efficiency is improved. The experiments are conducted in different kinds of environments and the output results demonstrate that the proposed system enables the robot to navigate efficiently and robustly in complex 3D environments.

## 1. Introduction

With the increasing aging population, the shortage of workforce has become one of the most challenging issues worldwide. Therefore, different kinds of robots have been attracting more and more interest in the past few decades—for example, the self-driving cars, warehouse robots, and wheelchair robots [1,2,3,4,5]. To perform a task autonomously in challenging environments, the mobile robot needs the ability to localize itself steadily, find the safe navigation path, and reach the desired position smoothly in unstructured environments.

**Figure 1:**
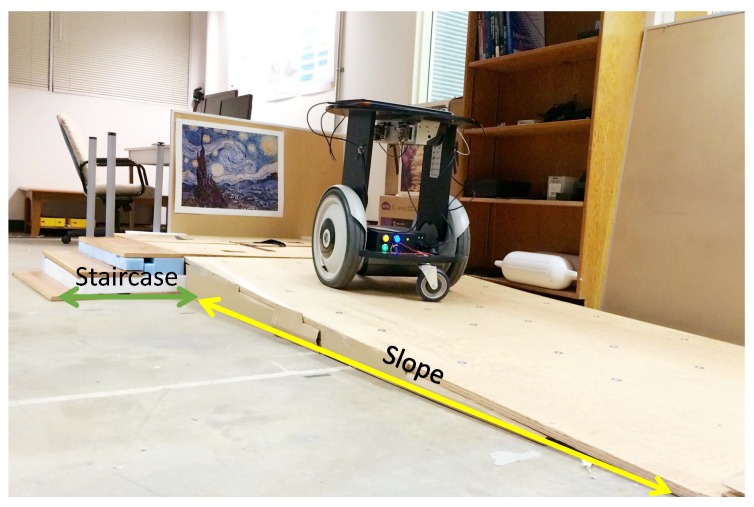
An illustration of a wheel robot navigating in a challenging environment.

Although many robots have shown the capabilities to navigate through uneven and cluttered environments, it is still an open problem to design an integrated system for autonomous robots navigation in unstructured indoor areas, especially in environments with narrow slope areas and cluttered space, which is the prerequisite for the service robot like the intelligent wheelchair robot. As shown in Figure 1, the robot is expected to safely navigate to a higher platform through narrow uneven areas while avoiding dynamic obstacles such as people and pets. The onboard navigation system should be capable of helping the robot localize itself in the environment robustly, distinguishing the slope area from the staircases, and outputting a safe path for the robot to navigate through.

This paper introduces an integrated system framework for autonomous mobile robot navigation in human–robot co-existing indoor environments. The contributions mainly lie in three aspects:

- We propose a novel 3D mapping approach to generate a 3D map and a traversable map for the robot navigation in the environments with uneven terrain.
- We leverage a camera re-localization method based on the random forest to improve localization performance in 3D indoor environments.
- We adopt a modified version of the RRT approach which can tune the step size adaptively for generating the global path, and the elastic band method is adopted for generating the local path.

Both the simulation and real-world experiments are conducted and the experiment results demonstrate that our present integrated framework enables the robot to navigate safely and robustly in complex 3D environments.

The following sections are introduced. In Section 2, a literature review of the work focusing on autonomous navigation is presented. The adopted hardware and software platform utilized in this work is illustrated in Section 3. The subsequent three Section 4, Section 5 and Section 6 provide case studies of autonomous navigation. The simulation and real-world experiment results are reported in Section 7. The final section draws conclusions and introduces future work.

## 2. Related Work

Building fully autonomous robots to assist people has been attracting considerable interest in the past few decades [6]. Recently, the perception, planning, and control techniques have gained great advancement, which benefits the development of the fully autonomous robot. Rhino [6] is a museum tour-guide robot, which is embedded with a distributed autonomous navigation software architecture and provides the human–robot interaction interface. Robox [7] is an autonomous mobile platform with multi-modal interaction capabilities, identified with a novel localization approach and a powerful obstacle avoidance scheme. The Jinny robot in [8] especially focuses on human–robot interaction and autonomous navigation. In different environments, the robot can choose the strategy correspondingly. However, there are still lots of challenges lying along the way towards building fully autonomous robots, and few existing integrated robot platforms focus on the safe and robust navigation in challenging environments. In the following, we will do the literature review in three aspects: environment representation, global localization, and path planning, which are highly related to our integrated system.

### 2.1. Environment Representation

The map (e.g., metric map [9], topological map [10], semantic map [11], and hybrid map [12]) encodes the information of the environments that the robot can use to distinguish different terrains, localize itself, and plan a safe trajectory. To build these maps, the ultrasound sensor and the laser range finder are frequently used. Oftentimes, these sensors are mounted on robot platforms to build a 2D map of the world. However, it is not sufficient to represent the 3D world just relying on one slice of the environment, especially when the robot is operating in an uneven and unstructured environment. Recently, sensors with 3D environment sensing capabilities have become increasingly common and much efforts have been dedicated to building a precise 3D map with these sensors [13,14]. For example, KinectFusion [15] stimulates the development of a vision-based 3D SLAM with low cost RGB-D sensors. In [16], a novel 3D SLAM algorithm adopting a 3D laser scanner is proposed. The world is represented by point clouds and the real-time interpretation and update of 3D data still pose great challenges for low power laptops, and these slow updates result in the robot either moving slowly or being in a potentially unsafe manner. In addition, the free and occupied spaces are not modeled clearly. To cope with this problem, a more compact 3D environment representation, the Octomap, is utilized for autonomous legged robot navigation [17]. The OctoMap is an efficient tool that provides a flexible 3D environment representation, which divides the whole space into free space, occupied space and unknown space. As an application example of the Octomap, Hornung et al. [2] divide the Octomap into multiple layers, in which the planning of the robot mobile base and the manipulator are performed on different map layers to improve planning efficiency. Motivated by this application, we propose to use the multi-layer maps to generate the traversable map from the Octomap to detect the uneven terrain.

### 2.2. Vision-Based Global Localization

Various sensors, such as the laser scanner, Ultra Wide Band (UWB), and stereo camera, have been widely used to cope with the indoor localization and re-localization problem, among which the vision sensors are some of the most versatile due to their flexibility and low-cost properties [18]. In this study, we adopt the camera re-localization scheme for localizing the robot in indoor environments. Over the past few decades, the local-feature based and keyframe based camera re-localization methods are traditionally investigated [19,20]. However, these methods are environment-dependent and have limited generalization abilities, thus they cannot be simply applied to a specific environment. In addition, due to the lack of metric information of RGB images, pixels’ depth information is required, which inevitably introduces environment noise.

Recently, rapidly developed machine learning methods have enabled great breakthroughs in a variety of computer vision tasks and lots of efforts have been made to apply the advanced algorithms in learning-based methods to tackle the camera-based re-localization problem [21,22]. In contrast to the traditional methods that use the unreliable depth information, the learning-based methods are not limited by the depth image. Random forest based methods [23,24] are among the state of the art. In these methods, the forest is trained to predict the camera location without feature tracking by using a training data set that consists of labeled RGB images. On the contrary, the recent deep-learning based methods train a Convolutional Neural Network (CNN) to recover the camera location from the RGB dataset [25]. However, these methods are usually inferior to the random forest based method in terms of localization accuracy in indoor environments [26]. In addition, most of these methods are only tested in a very small spatial extent and none of these machine learning based camera re-localization methods have been integrated into the real robot system.

### 2.3. Sampling-Based Path Planning

RRT is an efficient sampling-based path planner for large-scale or high-dimensional complex environments and there have been lots of efforts in the past few decades devoted to making RRT an efficient path planner [27,28]. Nearest Neighbor Search and Collision Checking are two time-consuming parts in RRTs. In [29], tedious collision checking is avoided in the process of finding a path. Only if the path to the goal is found will the path be checked to determine whether it is collision-free. Kuffner et al. [30] propose an RRT-connect method, which starts the exploration from both the start and the goal regions. Gammell et al. [31] extend the classical RRT* method to an informed one that generates the path in a heuristic way. However, there is insufficient research focusing on the path planning in uneven and unstructured environments using the sampling-based method.

To implement the sampling-based method in uneven environments, T-RRT [32] is proposed considering the transition probability during the tree extension. This method is useful in handling uneven terrain while it is built upon the mass-point robot model, and little attention has been paid to the motion planning, which is the trajectory for the robot to execute. Motivated by the T-RRT, the work in [33] presents an optimal path planning solution in complex cost spaces. However, the applications using the robot with non-holonomic constraints are not specially considered in that approach. Moreover, little attention has been paid to developing an integrated system framework towards robot navigation in uneven terrain.

## 3. Proposed Framework

**Figure 2:**
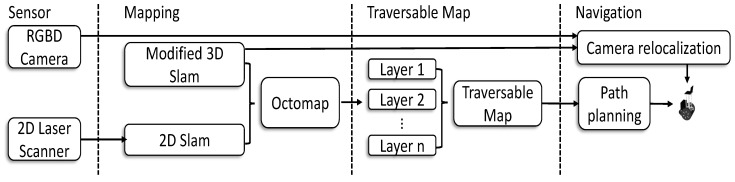
A schematic diagram of the system framework. The system uses a 2D laser scanner and an RGB-D camera. The mapping process outputs an OctoMap, which is cut into several layers to form a traversable map that is used for navigation purposes.

The software architecture of our proposed framework is illustrated in Figure 2. Both the laser data and the RGB-D data are the input for the 3D mapping module. A modified 3D SLAM method is proposed to generate the OctoMap, in which the 2D SLAM is utilized to increase the robustness of localization. With the proposed method, the generated OctoMap is cut into several layers to form a traversible for 3D navigation. To this end, we measure the differences between different layered maps and distinguish the staircase and slope accordingly. The generated traversable map indicates the collision-free area and the obstacle region, prompting the safe robot navigation in uneven environments. Furthermore, we propose to use a novel camera re-localization method based on the backtracking regression forest only using the RGB data as the input. The camera re-localization method ensures robust robot localization in 3D environments. Additionally, in our navigation framework, we use a novel path planning method based on our previous work in [34], which is more efficient than the standard RRT method. The generated global path is optimized by the local planner for the robot to execute. Details of our proposed framework are described in the following sections.

## 4. Environment Representation

In this section, we elaborate our proposed framework for environment representation, including a novel 3D mapping method and a traversable map generation approach.

### 4.1. 3D Environment Mapping

In order to generate a traversable map for 2D mobile robot navigation, a 3D map of the cluttered environment is built by means of a 3D SLAM scheme. The 3D SLAM pipeline is made up of two parts: mapping and localization. The localization part is critical for building a high-resolution map. In this work, we propose a hybrid localization paradigm by incorporating the 2D laser scan data and RGB-D data. The 2D laser data can bring robust localization performance in an environment without enough feature points, while the RGB-D data based visual odometry can perform better in feature-rich areas.

For environments that have rich feature points, we develop a SLAM method based on Oriented fast and Rotated Brief feature-SLAM (ORB-SLAM) [35]. In order to satisfy our need in the subsequent global localization and path planning procedures, we have two main differences compared with the original ORB-SLAM: more keyframes and 3D probabilistic OctoMap representation.

The SLAM module accounts for the 3D environment representation and training data generation for the global localization. Different from the ORB-SLAM which uses the survivalofthefittest strategy to select keyframes, the proposed architecture keeps more keyframes in order to obtain more camera poses for the training data during the camera re-localization. That is, we sacrifice the time spent on global pose optimization in SLAM to obtain more keyframe poses for more training data and more accurate global localization. Therefore, we utilize all the edges provided by the co−visibilitygraph [36] rather than using the essentialgraph in ORB-SLAM.

**Figure 3:**
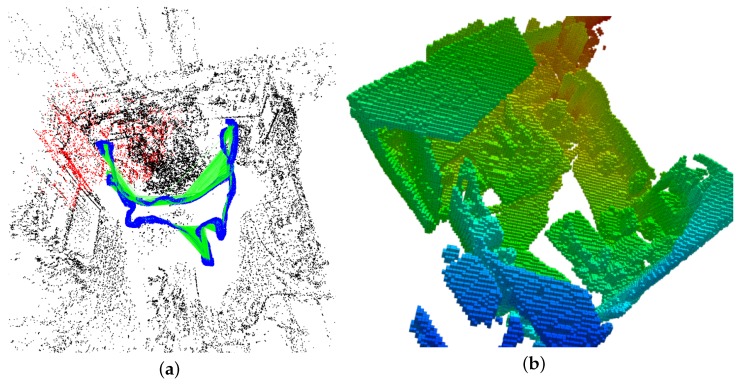
OctoMap representation for fr1/room of TUM RGB-D SLAM benchmark [37] with visual SLAM. (**a**) the sparse map from original ORB-SLAM [35], map points (**black**, **red**), keyframes (**blue**); (**b**) OctoMap representation.

As shown in Figure 3a, the output map from ORB-SLAM is too sparse to be directly used for path planning and navigation. To overcome this problem, we employ the camera pose of keyframes described above and the extracted point clouds as the input for the OctoMap to generate the 3D environment representation. Due to varying point densities, measurement error, and wrong registrations, the raw point cloud generated from RGB-D images or an RGB-D camera is affected by huge noises, posing great challenges for the subsequent surface/volume reconstruction. Therefore, an outlier-removal method based on the point distribution is employed to eliminate points with a big distance to its neighboring points. Then, the filtered point cloud is sent to the OctoMap and the result is shown in Figure 3b.

To further improve the robustness of the proposed 3D SLAM framework, a thread that performs 2D SLAM using a 2D laser scanner is set up to work along with the 3D SLAM process. The 2D SLAM can provide robust localization using a 2D occupancy grid map. The 2D localization result is fused with the localization part in 3D SLAM with the Extended Kalman Filter [38] that is widely used for the sensor fusion, to further improve the map quality. Implementation-wise, we adopt a software package that can take into account the localization results of both 2D SLAM and 3D SLAM and output a more accurate localization result.

### 4.2. Multilayer Maps and the Traversable Map

In this study, we specially focus on the distinguishment between the slope area (traversable) and the staircase (untraversable). On the basis of the OctoMap, we introduce the traversable map for the robot navigation in uneven environments. The traversable map is a kind of grid map that is extracted from the 3D OctoMap while it can represent the slope areas for the robot to navigate through. In contrast to the occupancy grid map, the states of the gird in the traversable map are divided by their traversability instead of by whether they are occupied or not.

**Figure 4:**
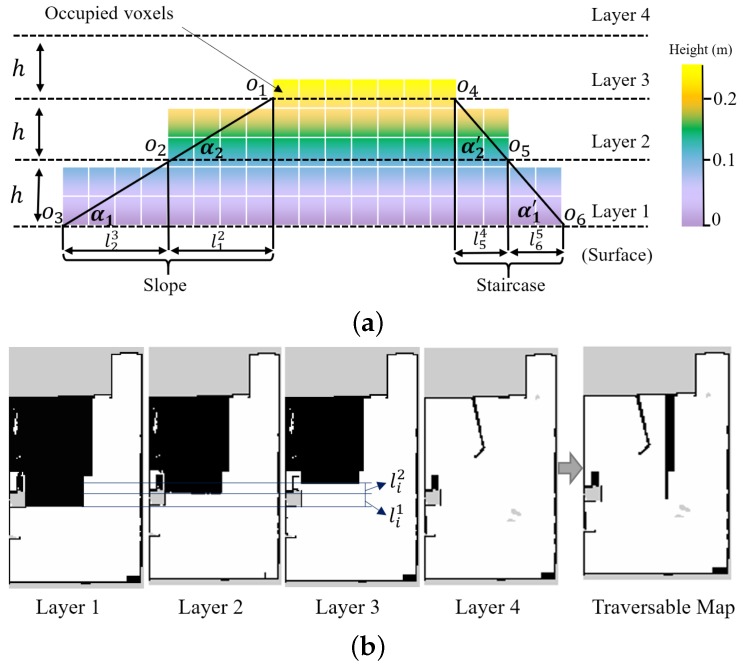
The schematic diagram of traversable map generation in a simulation environment. (**a**) occupied voxels representation of the slope and staircase. The colorful voxels represent the Octomap of the environment. (**b**) In the traversable map, the slope is marked as the collision-free area (**white area**) except the slope edge, while the staircase is marked as the occupied area (**black area**).

In order to get the traversable map from the 3D OctoMap, we proposed to cut the OctoMap into several layers and extract the useful information by comparing the differences between different layers. In this way, the slope area can be distinguished from the staircases. As shown in Figure 4, the colorful squares indicate the OctoMap of the environment. It is cut into several layers according to the distance *h*, which can be set to be as small as the resolution of the OctoMap. Viewed by the section image in Figure 4a, the OctoMap is cut into four layers: Layer 1 to Layer 4, in which the edge point on every layer map is indicated by $o_{i}$. The edge point $o_{i}$ and its adjacent edge point $o_{j}$ are used to measure the gradient of the terrain. The gradient angle $\alpha =arctan(h/l_{i}^{j})$, where $l_{i}^{j}$ is the horizontal distance between two adjacent edge points. If α is higher than a predefined threshold that is determined by the mobility of the robot, then the area is marked as untraversable, and vice versa. As shown in Figure 4a, for a slope that the robot can navigate through, $\alpha_{1}$ and $\alpha_{2}$ is less than the threshold. However, for a staircase, which can be regarded as the obstacle in the navigation process, $\alpha_{1}^{\prime }$ and $\alpha_{2}^{\prime }$ is higher than the threshold.

Figure 4b shows the layered map extracted from the OctoMap corresponding to the maps in Figure 4a and the generated traversable map. The edge on the layer can be detected by the edge detection method [39], where only the regular edges are considered in this illustrative example. In addition, the unknown regions are not taken into consideration in determining the terrain types. Layer 4 shows the map with a certain height that fewer obstacles are detected, as shown in Figure 4b. By utilizing Layer 1 to Layer 3 shown in Figure 4b and the aforementioned terrain detection method, the slope and the staircase can be detected effectively. The staircase, which is unfeasible for the robot to navigate through, is marked in Layer 4 to generate a navigable traversable map.

## 5. Global Localization

In this section, we will introduce the proposed global localization method based on the random forest. Compared with the localization method that uses the depth information, the proposed localization method based on RGB images is not prone to be affected by the illumination conditions and is more computationally efficient. The goal of camera-based re-localization is to predict the position of the camera based on the RGB image input. Through coordinate transformation, the position of the robot can be obtained consequently.

### 5.1. Random Forests Method

**Figure 5:**
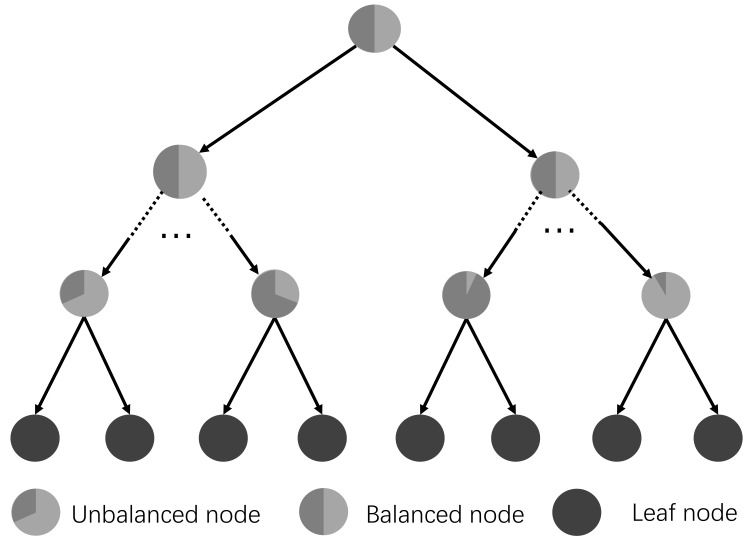
An example of a decision tree structure in the backtracking regression forest. The leaf nodes are depicted as a pie chart to illustrate the proportion of samples. More details can be found in [41].

In this study, the random forests based method is explored for the camera re-localization. In this method, the forests are trained to obtain the correspondences from the image pixels to the points in the 3D world coordinate directly and the correspondences are then used to predict the camera pose based on the RANSAC method [40]. In the training stage, each tree greedily splits the samples to minimize the spatial variance, which may cause unbalanced sub-trees, as shown in Figure 5. The reader could refer to [24] to obtain more details about the random forests. In our previous work [41], a novel sample-balanced objective and a backtracking scheme have been proposed to improve the prediction of camera pose. However, there is no specific model for the label on the leaf node, which makes the camera pose estimation method rely more on the training data that is sometimes difficult to acquire in real application cases. In this study, we further add a full-covariance Gaussian model in the leaf node. The point and line features are exploited in the regression forests to minimize the error of camera pose estimation. The key modules of the backtracking regression forest and the implementation details are introduced in the following.

### 5.2. Backtracking Regression Forest Training

**Image features**

The feature employed in the utilized forest is defined as:

(1) $$f_{\phi }\left(l_{p}\right)=I\left(l_{p},ch_{1}\right)-I\left(l_{p}+\frac{\delta }{D(l_{p})},ch_{2}\right),$$

where $f_{\phi }\left(l_{p}\right)$ is the pixel comparison feature [24,26], the term ϕ represents the feature response parameters δ: 2D offset; $ch_{1}$ and $ch_{2}:$ image channels. The term $I(l_{p},ch_{1})$ is the function that points out the RGB pixel lookup in channel $ch_{1}$. It is worth noting that sometimes the pixel depth cannot be obtained clearly, and we do not use these kinds of pixels for training and testing processes.

**Weak learner model**

In the tree shown in Figure 5, each split node *i* represents a weak learner parameterized by $\theta_{i}=\left\{\phi_{i},\tau_{i}\right\}$, where $\tau_{i}$ is a threshold. The tree grows recursively from the root node to the leaf node. For each separate node, the parameter $\theta_{i}$ is sampled from a set of randomly sampled candidates $\Theta_{i}$. More specifically, at each split node *i*, for the incoming training set $S_{i}$, samples are evaluated on split nodes to learn the split parameter $\theta_{i}$ that best splits the left child subset $S_{i}^{L}$ and the right child subset $S_{i}^{R}$ as follows:

(2) $$H\left(l_{p};\theta_{i}\right)=\left\{\begin{array}{cc} 0, & \;\mathrm{if}\;f_{\phi_{i}}\left(l_{p}\right)\leq \tau_{i},\;\mathrm{then}\;\mathrm{go}\;\mathrm{to}\;\mathrm{the}\;\mathrm{left}\;\mathrm{subset}\;S_{i}^{L},\\ 1, & \;\mathrm{if}\;f_{\phi_{i}}\left(l_{p}\right)>\tau_{i},\;\mathrm{then}\;\mathrm{go}\;\mathrm{to}\;\mathrm{the}\;\mathrm{right}\;\mathrm{subset}\;S_{i}^{R}. \end{array}\right.$$

The $\tau_{i}$ defines a threshold on feature $f_{\phi_{i}}\left(l_{p}\right)$. As proposed in Equation (1), we have adopted the pixel comparison feature. By introducing the more general weak learner model, the proposed framework can be applied in more applications.

**Training label**

The training set contains sequences of RGB-D frames with associated known camera poses P which includes 3×3 rotation matrix *R* and 3×1 translation vector *T* from the camera coordinate to the the world coordinate:

(3) $$P=\left[\begin{array}{cc} R & T\\ 0 & 1 \end{array}\right].$$

The 3D point x in camera coordinate of the corresponding pixel p could be computed by back-projecting the depth image pixels:

(4) $$x=\left[\begin{array}{c} x\\ y\\ z \end{array}\right]=\left[\begin{array}{c} \left(u-c_{x}\right)\times d/f_{x}\\ \left(v-c_{y}\right)\times d/f_{y}\\ d \end{array}\right],$$

where ${\left[u,v\right]}^{T}$ is the pixel p position in image plane, and ${\left[x,y,z\right]}^{T}$ is the point position in camera coordinate, ${\left[c_{x},c_{y}\right]}^{T}$ and ${\left[f_{x},f_{y}\right]}^{T}$ are the camera principal point and focal length, respectively. $d_{i}=depth_{image[v,u]}/factor$, and $depth_{image[v,u]}$ is the measured depth value at image point [v,u]. The scene’s world coordinate position m of the corresponding pixel p can be computed by:

(5) m=Px.

The associated camera pose P for each RGB-D image in the training data are obtained through camera tracking methods [15,35]. We utilize the ORB-SLAM to generate the training samples in the implementation. Image samples and the corresponding camera poses are simultaneously recorded in the environment to form the dataset.

**Training objective**

The information gain, $I_{i}$, is used as the utility function during the training phase. Here:

(6) $$I_{i}=E\left(S_{i}\right)-\sum_{j\in \{L,R\}}\frac{|S_{i}^{j}\left(\theta_{i}\right)|}{|S_{i}|}E\left(S_{i}^{j}\left(\theta_{i}\right)\right),$$

where $E(S_{i})$ is defined as the entropy of $S_{i}$, with $S_{i}$ represents a set of labels. $S_{i}^{j}\subset S_{i}$ is the set determined by the split parameter $\theta_{i}$. We proposed to use a single full-covariances Gaussian model to model the distribution of the labels $S_{i}$, so the entropy is defined as:

(7) $$E\left(S\right)=\frac{1}{2}log({\left(2\pi e\right)}^{d}|\Lambda \left(S\right)|),$$

with *d* is dimensionality and Λ represents the full covariance. This full-covariance Gaussian model follows the properties of Gaussian family, which brings the pose estimation problem into a probabilistic framework. Moreover, the full-covariance Gaussian mode lets every label follow a Gaussian separately, which will make the estimation process more accurate.

In the training process, the information gain is optimized that is:

(8) $$\theta_{i}^{*}=\arg\max_{\theta_{i}\in \Theta_{i}}I_{i}\left(S_{i},\theta_{i}\right).$$

For every node *i* on the random forest, it gets a random $\Theta_{i}$, as introduced in the weak learner model. Then, the parameter $\theta_{i}^{*}$ will be optimized by Equation (8). The parameter $\theta_{i}^{*}$ serves as the weak learner in the testing process.

After the training process, the samples reach the leaf nodes on the random forest. Since we employ a full-covariance Gaussian Model, each node on the forest has a mean vector and a covariance matrix. It is worth noting that, for each node, our model proposed to store a mean vector of local patch descriptors. Then, this descriptor is used to select the optimal predictions, as introduced in the following.

### 5.3. Regression Forest Prediction

In the testing phase, a regression tree greedily predicts 3D world coordinate positions by comparing the test sample feature values and the split values in internal nodes. When the sample arrives at a leaf node, the modes in that leaf node are the predictions. Since the comparison is conducted on a single dimension, it is inevitable to make mistakes. To alleviate this downside, a priority based backtracking strategy is utilized to locate the ideal forecast inside the time spending plan. More insights concerning the backtracking procedure can be found in [41].

In backtracking, the optimal mode has the minimum feature distance from the patch descriptor. Considering the computational efficiency and distinguish capability, we take advantage of a Walsh–Hadamard transform (WHT) to be the patch descriptor. Particularly, we use the first 20 Walsh–Hadamard projection vectors for each color channel in a 64×64 pixel patch.

#### Camera Pose Optimization

The backtracking regression forest described above is able to predict 3D world coordinate from any 2D image pixel. We use this 2D–3D correspondence to estimate the camera pose. The problem of estimating camera pose is formulated as the energy minimization:

(9) $$P^{*}=\arg\min_{P}E\left(P\right),$$

where P is a camera pose matrix. By minimizing the energy function, the camera pose can be estimated, thus the robot pose can be obtained according to the robot-camera coordinate transformation.

## 6. Planning and Navigation

We adopt the motion planning framework with an efficiently generated global path followed by a trajectory optimization process [42]. It is thus the prerequisite to get the global path efficiently. In this section, a variable step size RRT method is utilized to plan a global path in the uneven environment. Compared with the classical RRTs, the designed planner can construct a path from the start point to the goal point more efficiently. The global path ζ here contains a set of waypoints from the start position to the goal position without considering the motion constraint of the robot. Then, we use a local planner that continually generates a feasible path with the reference of the global path for the robot to execute. The local planner can also help the robot avoid the dynamic obstacles when it heads towards the goal.

### 6.1. Global Planner: Variable Step Size RRT

In our presented path planning approach, a local planner is used to optimize the generated sparse global path. Hence, the prerequisite is to build a path from the start point to the goal point in an efficient way. RRT can cope with path planning problems with complex constraints and obstacles and perform well in high dimensional environments. While in different environments, some parameters of this algorithm need to be adjusted properly in advance, among which one vital parameter is the step size for the RRT extension. We present a novel variable step size RRT for global path planning. In the following, we firstly provide an outline of the basic procedures of the classical RRT method.

Generally, the RRT is a tree structure that is built with collision-free nodes and edges in configuration space. A point $x_{r}$ is obtained randomly in the state space. It will search for the nearest point on the existing tree to get $x_{n}$, which is the nearest point to $x_{r}$. If the segment that connects $x_{n}$ to $x_{r}$ encounters no collision, then the point $x_{n}$ on the segment with limited step size to the point $x_{n}$, together with the collision-free edges, are added to the existing tree, which completes a tree extension process. This process repeats until the robot finds a connection from the start point to the goal point. Generally, the step size is required to be tuned for non-holomomic robots like Segway used in our experiment. When the step size is set to be small, it will take a longer time for the RRT to find the target. If the step size is set to be big, the generated path by RRT will jitter, which is not suitable for robot execution. Hence, it is vital to choose the most favorable step size according to the configuration space.

In this work, a novel RRT method is conceived that does not need to tune the step size. The algorithm is described in Algorithm 1. Firstly, following the rule of the standard RRT method, the function random(·) generates a random point $x_{r}$. Then, the function near(·) is applied to find the point on the existing tree T that is nearest to $x_{r}$. The major difference between our method and the standard RRT lies in the tree extension part. From the perspective of implementation, the step size is ignored in tree extension. $x_{n}$ and $x_{r}$ are connected with a segment. If the segment is collision free, then $x_{r}$ is added to the existing tree directly together with the segment. The whole process is indicated in Algorithm 1. In order to further improve the efficiency, an RRT-connect algorithm [30] is taken to build the tree from the start point to goal point concurrently. Once the two trees meet each other, the path between the start point and the goal point will be built.

**Algorithm 1:** Variable Step Size RRT

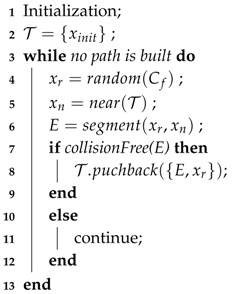



**Figure 6:**
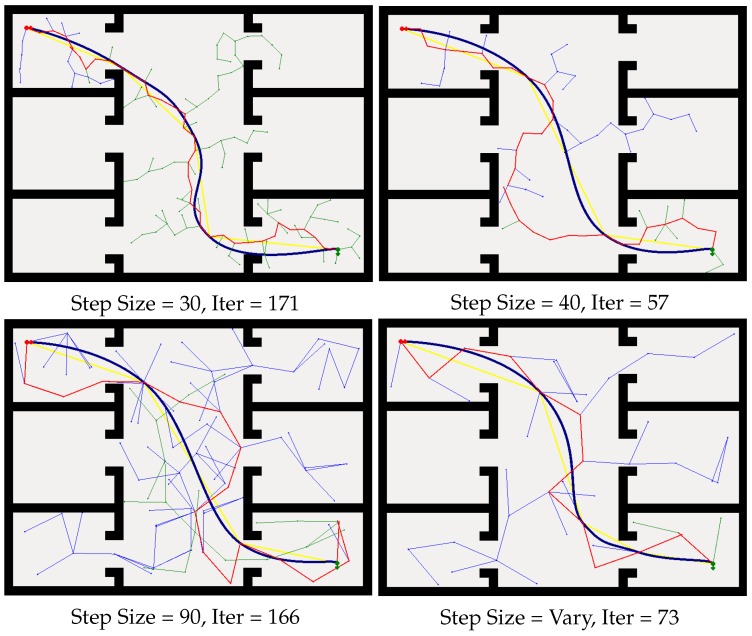
Path planning with limited step size RRT and our method. Blue line: RRT method. Red line: initial path form the start to the goal. Yellow and blue lines: optimization of the initial path.

As shown in Figure 6, with different step sizes, the RRT can build a path between the start point and the goal point. When the limitation of step size is set to be small, e.g., step size = 30, it iterates 171 times before it finds the path. When the step size is bigger, e.g., step size = 40, it iterates fewer times compared with the smaller step size, which means that longer step size will decrease the searching time for finding the path in the configuration space, while, if the step size is bigger, e.g., step size = 90, the searching path becomes jitter, and the searching iteration time is also large. Hence, we conclude that, for a certain environment, the step size is of great importance with limited step size RRTs. As shown in Figure 6, the proposed method can plan a path from the initial starting point to the target goal position efficiently. More significantly, our method does not need to preset the step size of RRT according to the experimental environments.

### 6.2. Local Planner and Path Execution

The global path planner generates a sparse path that cannot be used for non-holonomic robot navigation directly, as shown by the red line in Figure 6. This path is then modified by the elastic band method [43] to generate a series of control actions for the robot. Being the local planner, the elastic band method deforms the sparse path and outputs an optimal local path. The optimization process is shown by the yellow line and the blue curve in Figure 6. As the blue curve shows, the local path satisfies the non-holonomic motion constraints and hence can be executed by our robot. Details of the optimization mechanism of the elastic band can be found in [43]. In addition, the static and dynamic obstacles are taken into consideration in this process. The smooth path generated by the local planner is transformed into a set of control commands for the robot mobile base.

## 7. Experiments

### 7.1. Evaluation of Indoor Localization

To validate the performance of the camera localization framework, we conduct experiments using the 4 Scenes dataset [44]. We compare our proposed method with Perspective-n-Point (PnP) methods (SIFT+PnP, ORB+PnP) [45], the Random+Sparse method [46], and the MNG method [44], in the 4 Scenes dataset introduced by Valentin [44]. The main camera re-localization results are reported in Table 1. Our method using RGB-only images at testing time achieves higher accuracy than SIFT+PnP, ORB+PnP, and Random+Sparse, and is less accurate than the MNG method. However, the MNG method needs a large number of synthetic images for data augmentation, which is not required for our method. Moreover, the MNG method needs an explicit 3D model to render synthetic images to refine the pose, which is not mandatory in our method. According to the results, our method achieved better performance than all the baselines in camera re-localization accuracy when using RGB-D images at testing time.

### 7.2. Simulation Experiments

The simulation experiments are carried out to evaluate the effectiveness of our proposed framework including the localization method and the path planning method. The results are represented in the following two sections.

**Environment representation**

**Figure 7:**
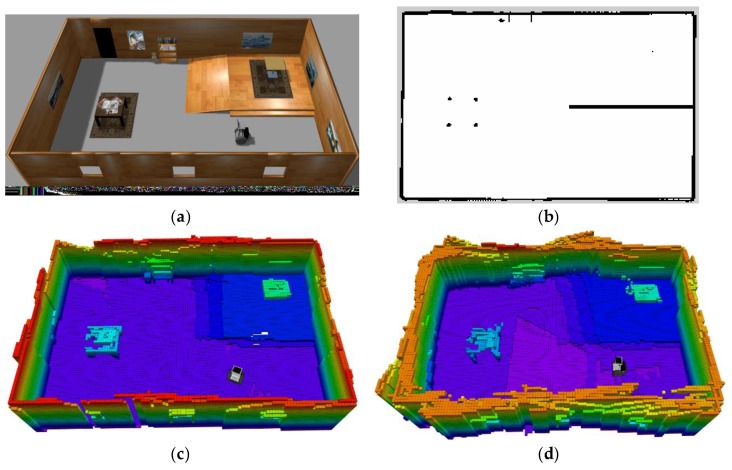
Traversable map generated from the pre-build OctoMap. (**a**) simulation environment; (**b**) traversable map; (**c**,**d**) OctoMap built by the 2D+3D SLAM method and singly 3D SLAM method.

In the simulation experiment, the environment is built in the Gazebo simulator, as shown in Figure 7a. The dimension of the simulated room is 16 m × 10 m × 3 m. The simulated robot model is equipped with one wheel odometry, a 2D laser range finder, and an RGB-D sensor. The 2D laser scanner is used to enhance the robot localization and an RGB-D sensor is used for the 3D SLAM. Figure 7c,d show the generated 3D OctoMap built by the proposed 2D+3D SLAM method and the 3D SLAM method, respectively. As shown in these figures, the proposed 2D+3D SLAM mechanism is more accurate in building the OctoMap than the method that only relies on the localization result of 3D SLAM. To make a compromise between the mapping efficiency and accuracy, the resolution of the map is set to 0.1 m/grid. Different colors in the OctoMap indicate different heights of the environment. It can be seen that the staircase area shows steeper changes than the slope area. Therefore, when generating the traversable map, the staircases are treated as untraversable area since their rate of change is larger, and the other areas are treated as traversable since their rate of change is lower than a threshold. Thus, we generate a traversable map, as shown in Figure 7c, which is used for our autonomous robot navigation.

**Autonomous navigation**

**Figure 8:**
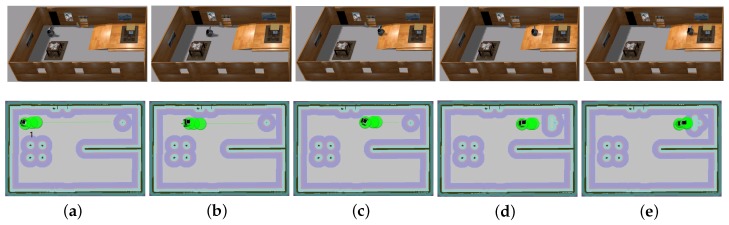
Simulation experiment of Task 1. The start location is indicated in (**a**). The robot is approaching the slope in (**b**,**c**). The robot starts to climb the slope in (**d**) and reaches the target above the ground in (**e**).

**Figure 9:**
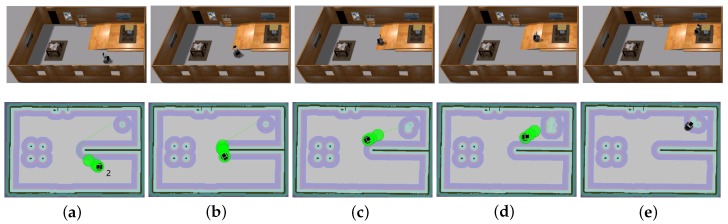
Simulation experiment of Task 2. The start location is indicated in (**a**). The robot is making a turn to approach the slope in (**b**,**c**). The robot climbs the slope in (**d**) and reaches the target above the ground in (**e**).

The traversable map serves as the input for path planning in uneven and unstructured environments and the global localization is achieved by means of the camera re-localization method. The target position is on a higher platform which can be reached from the ground via a slope or a staircase. In the proposed traversable map, the slope area is treated as the collision-free region while the staircase is treated as the obstacle. Two tasks with different start positions are designed for the robot, named Task 1 and Task 2, and the navigation results are shown in Figure 8 and Figure 9, respectively. In both figures, the first row shows images of autonomous navigation in Gazebo simulator, and the second row shows the Graphical User Interface (GUI) views of the process. The green lines show the sparse global path and the green bubbles show the local planner that optimizes the global planner and gives the robot control command.

We validate the efficiency of the proposed variable step size RRT method in Task 1. There are 20 experiments conducted to collect the data of the proposed RRT method and the traditional limited step size RRT method. The iteration and planning time are recorded in Table 2. For a specific environment, the step size needs to be preset for the limited step size RRT method. Inappropriate step size will make the global path jitter. On the contrary, our proposed method does not need to tune this parameter in advance and can provide a relatively smooth global path in a timely manner.

From Figure 8 and Figure 9, we can learn that the robot can navigate in the indoor environment with uneven terrain robustly. In Task 1, when the robot is placed in location 1 and the target is located in an area on the platform that is above the ground, it can use the traversable map to navigate robustly to the goal point. In Task 2, when the robot is placed in location 2 and the target is at the same localization with Task 1, the robot avoids the staircase and makes a turn to go to the target through the slope area. These two tasks demonstrate the capability of safe and robust navigation in indoor uneven terrain using our proposed framework. Details of these tasks are reported in Table 3. As specified by the table, the planner can plan a path efficiently and the robot can navigate through the unstructured environment with an acceptable velocity profile.

### 7.3. Real-World Experiments

**Figure 10:**
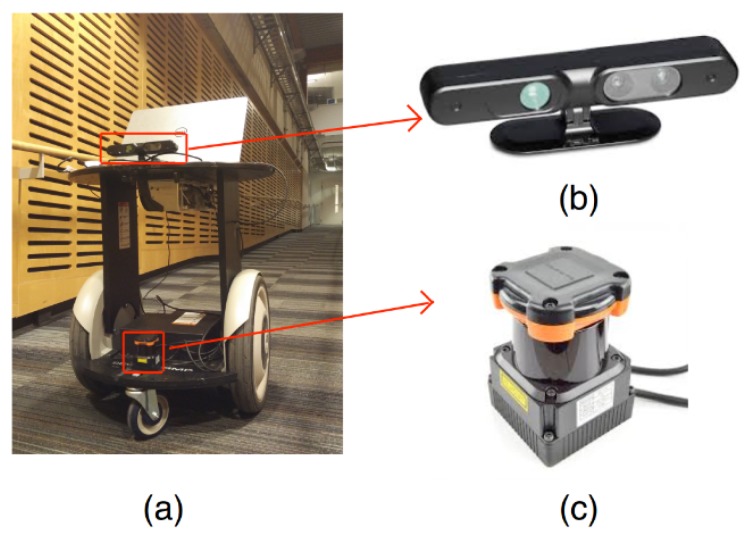
Robot hardware platform. (**a**) The Segway robot platform; (**b**) the Xtion RGB-D camera; (**c**) Hokuyo laser range finder.

**Figure 11:**
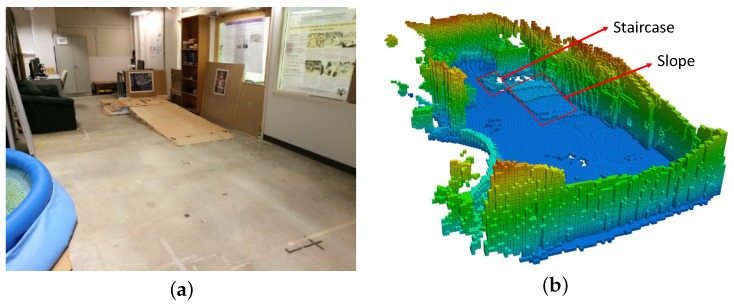
Real-word environment. (**a**) a snapshot of the real environmen; (**b**) the 3D representation of the environment with OctoMap, only occupied voxels are shown for visualization.

**Figure 12:**
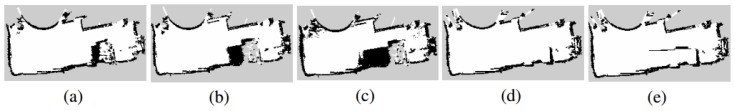
Multilayer maps and the traversable map for the real-world environment. (**a**–**d**) Multiple projected layers from OctoMap; (**e**) the traversable map. The staircases and slope edge are occupied while the slope is the free space.

To validate the performance of the integrated framework in real scenes, real-world environments are conducted with a Segway mobile robot platform, as shown in Figure 10. There is a Hokuyo laser scanner mounted on the mobile base and an Xtion RGB-D camera mounted on a higher plate. A Dell laptop on the high platform receives the sensor input and outputs the control command to the mobile base. The experimental environment is an uneven indoor environment with a staircase and a slope, as shown in Figure 11a. The OctoMap of the real-world environment generated by the modified 3D SLAM framework is shown in Figure 11b. The slope and the staircase can be straightforwardly distinguished with the OctoMap. The OctoMap is then cut into several layers to generate the traversable map, as indicated in Figure 12, which is then utilized for the robot navigation.

**Figure 13:**
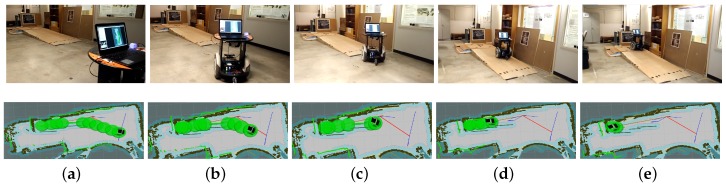
Robot autonomous navigation example in a real environment (Task 3 in Table 3). (**a**,**b**)The robot is approaching the slope; (**c**) the robot is to climb the slope; (**d**) the robot is climbing the slope; (**e**) the robot reaches the target above the ground.

**Figure 14:**
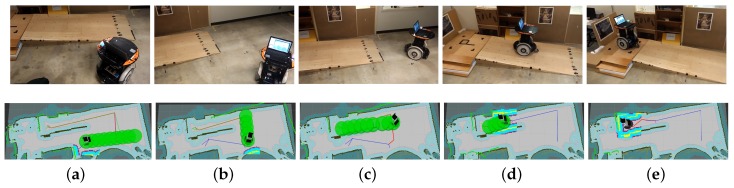
Robot autonomous navigation example in real environment (Task 4 in Table 3). (**a**,**b**) The robot is making a turn to approach the slope; (**c**) the robot is to climb the slope; (**d**) The robot is climbing the slope; (**e**) The robot reaches the target above the ground.

Similar to the above simulation environment setup, the target position in the real world is on a higher platform as well. Three representative task scenarios are designed for the experiment identified with three different start positions, named Task 3, Task 4, and Task 5. Table 3 shows the performance of the robot in each task. It could be found that, in Task 3, the robot can reach the target with 0.66 m/s and the path cost for this task is 8.3 m. The whole procedure is depicted in Figure 13. Differently, in Task 4, the robot is set to be near the target that is on a high platform above the ground, as shown in Figure 14. However, the staircase is unfeasible for the robot to go through. Figure 14 demonstrates the effectiveness of the proposed navigation scheme. The robot can avoid the staircase and reach the target through a slope. For conventional 2D localization methods, the robot tends to be lost when it makes a turn, while, in our case, the robot can localize itself robustly. Task 4 in Table 3 shows the statistics of our experiment. The robot moves a little slow since it makes more turns compared with Task 3 in this task.

**Figure 15:**
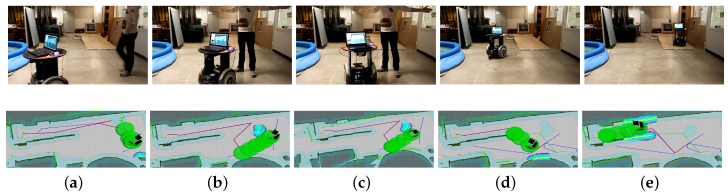
Dynamic obstacle avoidance (Task 5 in Table 3). (**a**) obstacle avoidance in the real scene; (**b**) the human suddenly blocks the way in front of the robot; (**c**) the robot changes direction to avoid the human; (**d**) the robot succesffully avoids the human; (**e**) the robot is climbing the slope.

To test the performance of obstacle avoidance of the proposed integrated framework, we leverage the local costmap to detect the obstacles in real time in the experiments. Different from the traversable map, the slope and staircase are not marked on the local cost map. Thus, these two objectives may be considered as obstacles. For example, if we set the local costmap to be the same size with our traversable map, then the slope may be marked as obstacles since it is above the ground and the laser may receive a reflected ray. In our implementation, the size of local costmap is set to be small enough, therefore the laser scan can be treated as an unanticipated ray. The robot can avoid the dynamic obstacles by the proposed approach. Facing a human that suddenly appears in the predefined path to the target, as Figure 15 indicates, the robot will replan its path to find another feasible path to the target. The average speed of this task is low for the safety, as Task 5 in Table 3 indicates.

## 8. Conclusions and Future Work

In this study, we solve the problem of autonomous robotic exploration in uneven and unstructured indoor environments. The proposed traversable map allows the robot to choose the slope area instead of the staircase to navigate to an expected target position. The proposed camera re-localization method based on a random forest tree can help localize the robot in the environment steadily. Moreover, the variable step size path planning method can plan a path on the traversable map efficiently. The following local planner proceeds to optimize the path to achieve safe navigation, which ensures that a mobile robot can move safely and robustly in complex 3D environment scenarios. As shown in the simulation and real-world experiments, the proposed hierarchical path planning framework is capable of generating a path efficiently. Furthermore, the proposed framework helps the robot move from its location to the expected target, which is higher above the ground.

The proposed integrated system can be widely used for many applications in the environment with uneven terrain, such as smooth navigation of wheelchair robots and complex disaster relief applications. Although our method can generate a traversable map for the robot to navigate through, some challenges still exist. We propose to cut the OctoMap into several layers to generate the traversable map, which is time-consuming and inaccurate in some complex environments. To solve this problem, in the future, we propose to use the RRT method to directly plan a path in the 2.5D space. By sampling points in the surface of the uneven terrain and checking the gradient of these segments, the RRT method can output a feasible path for the robot to follow.

## Figures and Tables

**Table 1:** Camera re-localization results for the indoor dataset compared with state-of-the-art methods.

	Frame Numbers		Spatial	Baselines				Our Results
Sequence	Training	Test	Extent	ORB+PnP	SIFT+PnP	Random+SIFT	MNG	
Kitchen	744	357	33 m${}^{3}$	66.39%	71.43%	70.3%	85.7%	**92.7**%
Living	1035	493	30 m${}^{3}$	41.99%	56.19%	60.0%	71.6%	**95.1**%
Bed	868	244	14 m${}^{3}$	71.72%	72.95%	65.7%	66.4%	**82.8**%
Kitchen	768	230	21 m${}^{3}$	63.91%	71.74%	76.7%	76.7%	**86.2**%
Living	725	359	42 m${}^{3}$	45.40%	56.19%	52.2%	66.6%	**99.7**%
Luke	1370	624	53 m${}^{3}$	54.65%	70.99%	46.0%	83.3%	**84.6**%
Floor5a	1001	497	38 m${}^{3}$	28.97%	38.43%	49.5%	66.2%	**89.9**%
Floor5b	1391	415	79 m${}^{3}$	56.87%	45.78%	56.4%	71.1%	**98.9**%
Gates362	2981	386	29 m${}^{3}$	49.48%	67.88%	67.7%	51.8%	**96.7**%
Gates381	2949	1053	44 m${}^{3}$	43.87%	62.77%	54.6%	52.3%	**92.9**%
Lounge	925	327	38 m${}^{3}$	61.16%	58.72%	54.0%	64.2%	**94.8**%
Manolis	1613	807	50 m${}^{3}$	60.10%	72.86%	65.1%	76.0%	**98.0**%
**Average**	—	—	—	53.7%	62.2%	59.9%	69.3%	**92.7**%

**Table 2:** Demonstration of the efficiency of the proposed variable RRT method.

Step Size	Iterations	Computational Time (ms)	Jitter
30	165	12	0/20
40	63	4	0/20
50	92	7	4/20
60	113	9	8/20
70	147	10	12/20
Variable	67	4	0/20

**Table 3:** Statistics of the speed, distance, and planning time during the experiments.

Tasks	Speed (ave) (m/s)	Traveled Distance (m)	Planning Time (ms)
**Simulated environments**			
Task 1	0.58	12.1	3.9 ± 2
Task 2	0.42	6.0	6.7 ± 2
**Real-world environments**			
Task 3	0.66	8.3	8.0 ± 2
Task 4	0.38	9.0	12.5 ± 2
Task 5	0.35	7.0	10.0 ± 2
